# Hypermethylation of *Bmp2* and *Fgfr2* Promoter Regions in Bone Marrow Mesenchymal Stem Cells Leads to Bone Loss in Prematurely Aged Mice

**DOI:** 10.14336/AD.2024.0324

**Published:** 2024-03-24

**Authors:** Yao Wang, Lin Sun, Tianyou Kan, Wendong Xue, Han Wang, Ping Xu, Lei Zhang, Mengning Yan, Hanjun Li, Zhifeng Yu

**Affiliations:** ^1^Shanghai Key Laboratory of Orthopedic Implants, Department of Orthopedic Surgery, Shanghai Ninth People's Hospital, Shanghai Jiao Tong University School of Medicine, Shanghai, China.; ^2^Shanghai Jihui Laboratory Animal Care Co., Ltd., Shanghai, China.; ^3^Department of Oral & Cranio-Maxillofacial Surgery, Ninth People's Hospital, Shanghai Jiao Tong University School of Medicine, Shanghai, 200011, PR China.; ^4^Clinical Stem Cell Research Center, Ren Ji Hospital, Shanghai Jiao Tong University School of Medicine, Shanghai, China

**Keywords:** Osteoporosis, DNA methylation, Epigenetics, BMSC, Osteogenesis

## Abstract

Osteoporosis is an age-related, systemic skeletal disease that poses a significant public health challenge in contemporary society. Development at the epigenetic level is emerging as an important pathogenic mechanism of osteoporosis. Despite indications of a robust association between DNA methylation and osteoporosis development, a comprehensive understanding of the specific role of DNA methylation in osteoporosis remains limited. In this study, significant bone loss was detected at the beginning of eight weeks of age in mouse models of premature aging (SHJH*^hr^* mice). We identified a notable upregulation of DNA methyltransferase 3b/3l (*Dnmt3b/l*) and downregulation of ten eleven translocation dioxygenase 1 (*Tet1*) in bone marrow mesenchymal stem cells (BMSCs) isolated from SHJH*^hr^* mice, along with an increase in the overall 5-methylcytosine (5mC) levels. Moreover, methylation capture sequencing revealed genomic hypermethylation in SHJH*^hr^* mice BMSCs. Integrated methylome and transcriptome analyses revealed several crucial methylated genes and networks that are potentially associated with osteoporosis development. Notably, elevated methylation levels of genes linked to the Wnt signaling pathway, particularly bone morphogenetic protein 2 (*Bmp2*) and fibroblast growth factor receptor (*Fgfr2*), appeared to compromise the osteogenic differentiation potential of BMSCs. Concurrently, DNA methyltransferase inhibitors attenuated the methylation of the promoter regions of *Bmp2* and *Fgfr2* and rescued the osteogenic differentiation potential of the BMSCs from SHJH*^hr^* mice. In summary, our study provides novel insights into the role of DNA methylation in the development of osteoporosis and suggests promising prospects for employing epigenetic interventions to manage osteoporosis.

## INTRODUCTION

Osteoporosis significantly increases the risk of pathological fractures, which can result in disabilities. Senile osteoporosis, which is the most prevalent form of primary osteoporosis, increases with age [1]. An imbalance between osteoclast-mediated bone resorption and osteoblast-mediated bone formation is the primary cause of osteoporosis. Pro-inflammatory factors generated by senescent cells, which contribute to senescence-associated secretory phenotypes (SASPs), disrupt the skeletal microenvironment. The SASPs contribute to a declined osteoblast and increased osteoclast function [2, 3]. Most notably, the senescence of bone marrow mesenchymal stem cells (BMSCs) contributes significantly to osteoporosis [4]. With age, the osteogenic differentiation capacity of BMSCs decreases, whereas adipogenic differentiation capacity increases, often leading to osteoporosis accompanied by the accumulation of bone marrow adipose tissue (BMAT) [5]. In summary, osteoporosis mainly results from an imbalance between osteoblast and osteoclast functioning, which are influenced by various factors. Recent research has shown that the disruption of epigenetic information, including DNA methylation, is a prominent cause of aging onset. Addressing these changes can potentially improve the mammalian aging phenotype [6], suggesting the feasibility of epigenetic intervention strategies for osteoporosis.

As an important epigenetic modification, DNA methylation significantly influences aging [7]. DNA methylation and demethylation are primarily orchestrated by the DNA methyltransferase (*Dnmt*) and ten eleven translocation dioxygenase (*Tet*) family, respectively [8]. *Dnmt* and *Tet* genes play crucial roles in bone metabolism and development. *Dnmt3a*-mediated methylation of anti-osteoclastogenic genes facilitates osteoclast maturation. Specific *Dnmt3a* knockdown in osteoclasts resulted in increased bone density in mice [9]. Wang et al. demonstrated that Tet proteins interact with runt-related transcription factor 2 (*Runx2*) through their catalytic structural domains to regulate cytosine methylation around the binding region of *Runx2*, highlighting the impact of *Tet* genes on bone development [10]. DNA methylation, an epigenetic regulation mechanism of gene expression, plays a pivotal role in regulating the Wnt signaling pathway [11, 12]. For example, in lupus erythematosus osteoporosis, the upregulation of *Dnmt1* results in hypermethylation of the promoter region of *Notch1*, resulting in the inhibition of Notch signaling and promotion of Wnt signaling, thus enhancing BMSC differentiation into osteoblasts [13]. DNA methylation significantly influences bone metabolism and development, suggesting its potential role in the regulation of skeletal aging.

In our previous study, we identified a mouse model that aged prematurely (SHJH*^hr^*) with accelerated skin aging at 4 weeks [14]. A recent study conducted by Liang et al. revealed that skin aging over time may contribute to age-related bone loss, suggesting a correlation between skin atrophy and bone loss with age [15]. In this study, we used SHJH*^hr^* mice as the animal models for premature aging and found that their bone mass reduced significantly after eight weeks of age. Further, the senescence index of the BMSCs from SHJH*^hr^* mice was upregulated, while osteogenic capacity decreased. Additionally, *Dnmt3b*/*l* in the BMSCs of prematurely aged SHJH*^hr^* mice upregulated significantly, accompanied by an increase in overall 5-methylcytosine (5mC) levels. We uncovered the signaling pathways and key genes regulated by DNA methylation through comprehensive methylome and transcriptome analyses, with the aim of identifying new targets for epigenetic intervention in osteoporosis.

## MATERIALS AND METHODS

### Animals

Male ICR mice were provided by Shanghai Jihui Laboratory Animal Care Co., Ltd. (Shanghai, China). The mice were raised under a 12-hour light/dark cycle and allowed free access to food and water. For the treatment experiment, we observed the effects of SGI-1027 (Cat#: HY13962, MCE) on SHJH*^hr^* mice *in vivo* by using 12-week-old male mice for animal experiments. SGI-1027 is stored in powder at -20 °C.SGI-1027 was injected into mice for two weeks (five days per week, intraperitoneal injection at 2.5 mg/kg). All animals used in this study received ethical approval and care in accordance with the institutional guidelines developed by the Animal Experiment Ethics Committee of Shanghai Jiao Tong University School of Medicine (No. SH9H-2023-A858-1).

### Immunohistochemistry stain

Bone samples were immersed in 4% paraformaldehyde (Cat#: P0099, Beyotime) at 4 °C for 24 h and decalcified in 10% EDTA (Cat#: BL616A, Biosharp) at 4 °C for 4 weeks and then embedded in paraffin. Formaldehyde-fixed, paraffin-embedded bone tissue sections (5 μm) were assessed using immunohistochemical (IHC) staining. The sections were deparaffinized and hydrated with distilled water, followed by antigen recovery using 0.25% trypsin. Endogenous peroxidase activity was blocked using hydrogen peroxide. Sections were incubated with primary antibodies against Bmp2(Cat#: A0231, Abclonal, 1:200), Dnmt3b (Cat#: A11079, Abclonal, 1:200), tet1(Cat#: A1506, Abclonal, 1:200) and Dnmt3l (Cat#: A2342, Abclonal, 1:200) overnight at 4 °C, followed by incubation with horseradish peroxidase-conjugated secondary antibodies. Bmp2, Dnmt3b, TET1, and DNMT3L positivity rates in the stained areas were calculated using Image-Pro Plus software (Media Cybernetics, Rockville, MD, USA).

### Micro-CT analysis

Right tibias were scanned using a high-resolution μCT scanner (μCT 80; Scanco, Zurich, Switzerland) to obtain the trabecular and cortical bone microstructure (BV/TV, Tb.N, Tb.Sp, Conn.D, Ct.Th, and SMI). The scanning parameters were set as follows: voltage, 70 kV; electric current, 114 μA; and resolution, 10 μm per voxel. For trabecular measurements, a region of interest was defined as 1.9 mm from the proximal tibial condyles, immediately distal to the growth plate, and extended to 100 slices.

### Three-point bending tests

Three-point bending tests were performed on the mid-shaft of fresh right femoral bones obtained from all mice, using a model 3366 Dynamic Mechanical Analyzer (Instron, Norwood, MA, USA).

### Cell culture

BMSCs were harvested from the bone marrow of ICR mice. Bone marrow cells from mouse femurs and tibiae were washed, collected in T25 flasks, and incubated overnight at 37 °C in a 5% CO2 incubator. The cells were then rinsed twice with phosphate-buffered saline (PBS) to remove the unadhered cells. The adherent cells were kept in a medium containing 10% fetal bovine serum (FBS) (Cat#: 16000-04, Gibco) and 1% penicillin and streptomycin (PS) (α-minimum essential medium (α- MEM). For cultivating BMMs, extract bone marrow cells and place them in MEM-α culture medium containing fresh 10% fetal bovine serum (FBS) containing 25ng/mLMcsf. Observe the cell status every 2-3 days and replace the culture medium. For the induction of osteoclasts, we induced BMMs using a culture medium containing 25ng/mLMcsf (Cat#: RP01216, abclonal) +50ng/mLRankl (Cat#:462-TEC-010, R&D Systems). After 5-7 days, the stimulation was terminated by observing the fusion of osteoclasts. Regarding TRAP staining, after fixing the cells, prepare TRAP staining solution according to the instructions of the Sigma TRAP staining kit. After co-culturing at 37 ° C in the dark for 30-45 minutes, image acquisition can be performed.

### Alizarin red staining

Cells were cultured at 37 ° C and 5% CO2 until 60-70% confluence. The supernatant was discarded and added to osteogenic differentiation medium (Cat #: muxmx-90021, Oricelll, China). Change the osteogenic culture medium every 2-3 days and culture at 37 ° C and 5% CO2 for approximately 21 days to observe changes in cell morphology. Terminate induction is based on the precipitation of calcium salt crystals and the formation of calcium nodules. Staining and identification: Cells were washed twice with PBS, fixed at room temperature with 4% paraformaldehyde for 15 minutes, washed twice with PBS, stained with Alizarin Red staining solution (Cat #: muxmx-90021, Oricelll, China) at room temperature for 30 minutes, washed twice with PBS, and observed under a microscope.

### Alkaline phosphatase staining

Cellular induction was terminated after seven days as described previously. For staining identification, the cells were washed twice with PBS, fixed with 4% paraformaldehyde for 15 min at room temperature, washed twice with PBS, stained with alkaline phosphatase staining solution (Cat #: C3250S, Beyotime, China) for 30 min at room temperature, re-washed twice with PBS, and visualized under a microscope.

### Senescence-associated β-galactosidase (SA-β-Gal) staining

For BMSCs cultured in six-well plates, the culture medium was aspirated, and the cells were washed once with PBS, to which 1 mL of fixative was added. The cells were fixed for 15 min at room temperature; the fixative was aspirated; and the cells were washed with PBS three times for 3 min each time. The PBS was then removed, and 1 mL of SA-β-gal staining solution (Cat#: C0602, Beyotime) was added to each well. The wells were sealed with plastic wrap to prevent evaporation of the staining solution and incubated at 37 °C overnight. After incubation, the staining solution was removed, 2 mL PBS was added, and images were captured using a light microscope (Olympus, Japan).

### Immunofluorescence (IF) assay

BMSCs were fixed with 4% paraformaldehyde for 30 min and blocked with 5% goat serum for 1 h, followed by incubation with anti-P16 (Cat#: 10883-1-AP, Proteintech,),anti-P21 (Cat#: 2947, CST, 1:100), anti-γH2AX (Cat#: AP0687, abclonal, 1:100), anti-Ki67 (Cat#: A25399, Abclonal; 1:100), overnight at 4 °C. BMSCs were incubated with Alexa Fluor® 555 goat anti-rabbit IgG (Cat#: A21428a, Invitrogen, 1:150) for 30 min at RT. The nucleus was stained with DAPI. The results were observed under a Leica TCS SP5 laser confocal scanning microscope (Mannheim, Germany).

### EdU staining

All reagents in this experiment are from the reagent kit (Cat#: C0071S, Beyotime, China). Prepare EdU culture medium according to the requirements of the reagent kit and add it to cells for co-cultivation for 2 hours. After cleaning the cells, add 4% paraformaldehyde (30min) and 0.5% TritonX-100(10 min) successively and rinse the cells three times. Then add endogenous peroxidase blocking solution and incubate at room temperature for 20 minutes. After cleaning the cells, prepare CLICK reaction solution according to the requirements of the reagent kit. Add CLICK reaction solution to the cells and incubate for 20 minutes. After cleaning the cells, add Streptavidin-HRP working solution and incubate for 30 minutes. Wash the cells after finishing. Add DAB colorimetric solution and incubate for 20 minutes. Wash the cells after finishing and add DAPI staining solution and stain for 10 minutes. Wash the cells and observe under a Leica TCS SP5 laser confocal scanning microscope (Mannheim, Germany).

### RNA-sequencing (RNA-Seq)

TRIzol (Life Technologies, Carlsbad, CA, USA) was used to isolate total RNA from the third generation of the two groups of BMSCs. RNA-Seq library construction and high-throughput RNA sequencing were performed by Shanghai Ouyi Biomedical Technology Co., Ltd. (Oebiotech, China) using a BGISEQ-500 high-throughput sequencer.

### 5mC determination

After DNA quantification, methylation analysis of the same amount of genomic DNA was performed using a mouse 5-mc ELISA Research Kit (Cat#: RC-E108723A Ruichuang, China). In short, the kit uses a dual antibody one-step sandwich enzyme-linked immunosorbent assay (ELISA). Add cell supernatant, standard substance, and HRP labeled detection antibody to the micropores pre coated with 5-methylcytosine (5-mC) antibody, then incubate and thoroughly wash. Using substrate TMB for color development, TMB is converted to blue under the catalysis of peroxidase, and finally to yellow under the action of acid. The depth of color is positively correlated with the presence of 5-methylcytosine (5-mC) in the sample. Measure the absorbance (OD value) using an ELISA reader at a wavelength of 450nm and calculate the sample concentration.

### MeDIP-seq

MeDIP-seq was performed by Shanghai Jingzhou Gene Technology Co., Ltd., China. Briefly, DNA was sheared using a Covaris S2 system, and the target DNA peak size was 250 bp. Thereafter, 3ʹ ends were repaired and adenylated. After adaptor ligation, the beads were used for fragment selection. Subsequently, methylated DNA immunoprecipitation was performed, and the eluted DNA was amplified by PCR to form the final sequencing library. The PCR products were purified, and library quality was assessed using an Agilent Bioanalyzer 2100. Finally, paired-end sequencing was performed using an Illumina NovoSeq6000.

**Table 1:** Primer sequence (5ʹ to 3ʹ).

Gene	Forward	Reverse
***Fgfr2***	GCTATAAGGTACGAAACCAGCAC	GGTTGATGGACCCGTATTCATTC
***Bmp2***	GGGACCCGCTGTCTTCTAGT	TCAACTCAAATTCGCTGAGGAC
***Tet1***	ACACAGTGGTGCTAATGCAG	AGCATGAACGGGAGAATCGG
***Tet2***	CTTACCACCCATCCACACCC	AGCTATAGCCACCCCTCCAA
***Tet3***	TCCGGGAACTCATGGAGGAT	GTCGATCGCCACATCCTCAT
***Dnmt1***	AAGAATGGTGTTGTCTACCGAC	CATCCAGGTTGCTCCCCTTG
***Dnmt3a***	GAGGGAACTGAGACCCCAC	CTGGAAGGTGAGTCTTGGCA
***Dnmt3b***	AGCGGGTATGAGGAGTGCAT	GGGAGCATCCTTCGTGTCTG
***Dnmt3l***	CCTCCTCCAATCTCAGATGC	AATCGGCTAGAAGGGTCTGC
***Ocn***	CTGACCTCACAGATCCCAAGC	TGGTCTGATAGCTCGTCACAAG
***Opn***	ATCTCACCATTCGGATGAGTCT	TGTAGGGACGATTGGAGTGAAA
***Gapdh***	ACCCAGAAGACTGTGGATGG	CACATTGGGGGTAGGAACAC
***Acp5***	CACTCCCACCCTGAGATTTGT	CATCGTCTGCACGGTTCTG
***Ctsk***	GGACCCATCTCTGTGTCCAT	CCGAGCCAAGAGAGCATATC
***Mmp9***	CGCCACCACAGCCAACTATGAC	CTGCTTGCCCAGGAAGACGAAG
***Atp6v0d2***	TTCCTTGGAGCCCCTGAGCACAT	TGTGAAACGGCCCAGTGGGTG
***Sema5a***	GACTTGCTAGGCCCGAGAC	TCTGAACTCCCGTAACCAGGG

### RNA extraction and RT-qPCR

Total RNA was extracted from the cultured cells using the AxyPrep Multisource RNA Miniprep Kit (Cat#: AP-MN-MS-RNA-250, Axygen, Corning, New York, USA). Briefly, after the cells were lysed, centrifugation and washing were performed using various reagents from the kit. Finally, TE buffer was added to dissolve the RNA, which was then centrifuged to obtain a liquid RNA sample. Complementary DNA (cDNA) was synthesized by reverse transcription using TaKaRa Reverse Transcription Reagent (Cat#: D2680A, TaKaRa, Japan). RT-qPCR was performed using a QuantStudio 6 Flex RT-qPCR System (Applied Biosystems, CA, USA) and SYBR Green PCR Mix (Cat#: B21402, Bimake, TX, USA). The relative RNA level was calculated using the comparative threshold cycle (2^-ΔΔCT^) method and normalized to *Gapdh* value within the sample. The primers used in this study are shown in the Table. 1.

### Western blot analysis

For the extraction of total proteins, we lysed the cells for 15 min using cell lysis buffer (Cat#: P0013C, Beyotime, China) containing protease inhibitors (Cat#: P1005, Beyotime, China) and then sonicated the cells. The collected protein solution is mixed with Sampling Buffer (Cat#: P0015, Beyotime, China) and incubated at 99°C for 10 minutes. Proteins were then separated with 4-20% ExpressPlus™ PAGE Gel (Cat#:M01215C, GenScript) and electrophoresed in Tris-MOPS-SDS Running Buffer (Cat#:M00677, GenScript) diluted with ddH2O. The gels were then electroblotted onto 0.22 µm PVDF membranes (Cat#: L00735, GenScript). Gels were blocked with 5% BSA-TBST (Tris-buffered saline (TBS)-0.1% Tween 20) (Cat#:ST673, Beyotime, China) for 1 h at room temperature. The membrane was incubated with primary antibodies overnight at 4 °C. The primary antibodies used were anti-Bmp2 antibody (Cat#: A0231,Abclonal, 1:1000), anti-Runx2 antibody (Cat#: GTX119505, Genetex, 1:500), anti-Ocn antibody (Cat#: 16157-AP, Proteintech, 1:1000), anti-Opn antibody (Cat#: 22952-AP, Proteintech, 1:1000), anti-γH2AX antibody (Cat#: AP0687, abclonal, 1:1000), anti-p21 antibody (Cat#: 2947, CST, 1:1000), anti-p16 antibody (Cat#: 10883-1-AP, Proteintech, 1:500), anti-Dnmt3b antibody (Cat#: A11079,Abclonal, 1:1000), anti-Dnmt3l antibody (Cat#: A2342,Abclonal, 1:1000), anti-Tet1 antibody ((Cat#: A1506,Abclonal, 1:1000), anti-Fgfr2 antibody (Cat#: ab109372, abcam, 1:500) and anti-Gapdh antibody (Cat#: 60004-1-Ig, Proteintech, 1:50000). The next day, the membranes were washed with PBS and then incubated with an appropriate secondary antibody (Cat#: SA00001-1 or SA00001-2, Proteintech, 1:5000) in 2% non-fat milk for 1 h. The immunoreactive bands were detected using a chemiluminescence kit (Cat#: RPN2232, Amersham Biosciences Ltd., UK) and then analyzed using Image-Pro Plus software (version 6.0). The relative protein expression level was normalized to the intensity of the Gapdh band.

### Methylation-specific PCR

Methylation-specific PCR (MSP) primers were designed for sites where the *Bmp2* and *Fgfr2* promoter regions were methylated by methylation sequencing. The cells were treated using an animal tissue/cellular genomic DNA extraction kit (Cat#: DP304, TIANGEN, Beijing, China) and a sulfite transformation kit (Cat#: D0068S, Beyotime, China). The treated DNA fragments were amplified and purified by gel electrophoresis.

### Cell Counting Kit-8 (CCK-8) cytotoxicity assay

For the cytotoxicity assay, the cells were cultured in 96-well plates at a concentration of 5 × 10^3^ cells/well for 24 h. Subsequently, cell proliferation was assessed using a CCK-8 assay (Cat#:C0037, Beyotime, China). Absorbance (mean optical density) at 450 nm was measured using an Infinite M200 Pro multimode microplate reader (Tecan Group, Ltd., Männedorf, Switzerland).

### Statistical analysis

Data are shown as mean ± standard deviation (SD), and the results are represented as bar graphs with individual data points. The color intensity of the images was measured using ImageJ v1.8.0 software (National Institutes of Health, USA). Statistical analyses were performed using Prism Version 9 software (GraphPad, California, USA) and SPSS22.0 (IBM, New York, NY, USA). All data were tested for normality using the Shapiro-Wilk test. An unpaired two-tailed Student's t-test was used for pairwise comparisons between two groups. For comparisons involving three or more groups, a one-way analysis of variance (ANOVA) followed by Tukey’s post-hoc test was used. The non-parametric Mann-Whitney U test was used to compare non-parametric datasets (non-normal distribution or n<6) between two groups. P ≤ 0.05 was considered a statistically significant difference.

## RESULTS

### Prematurely aging mice displayed a notable decline in bone mass at 8 weeks

**Figure 1.**
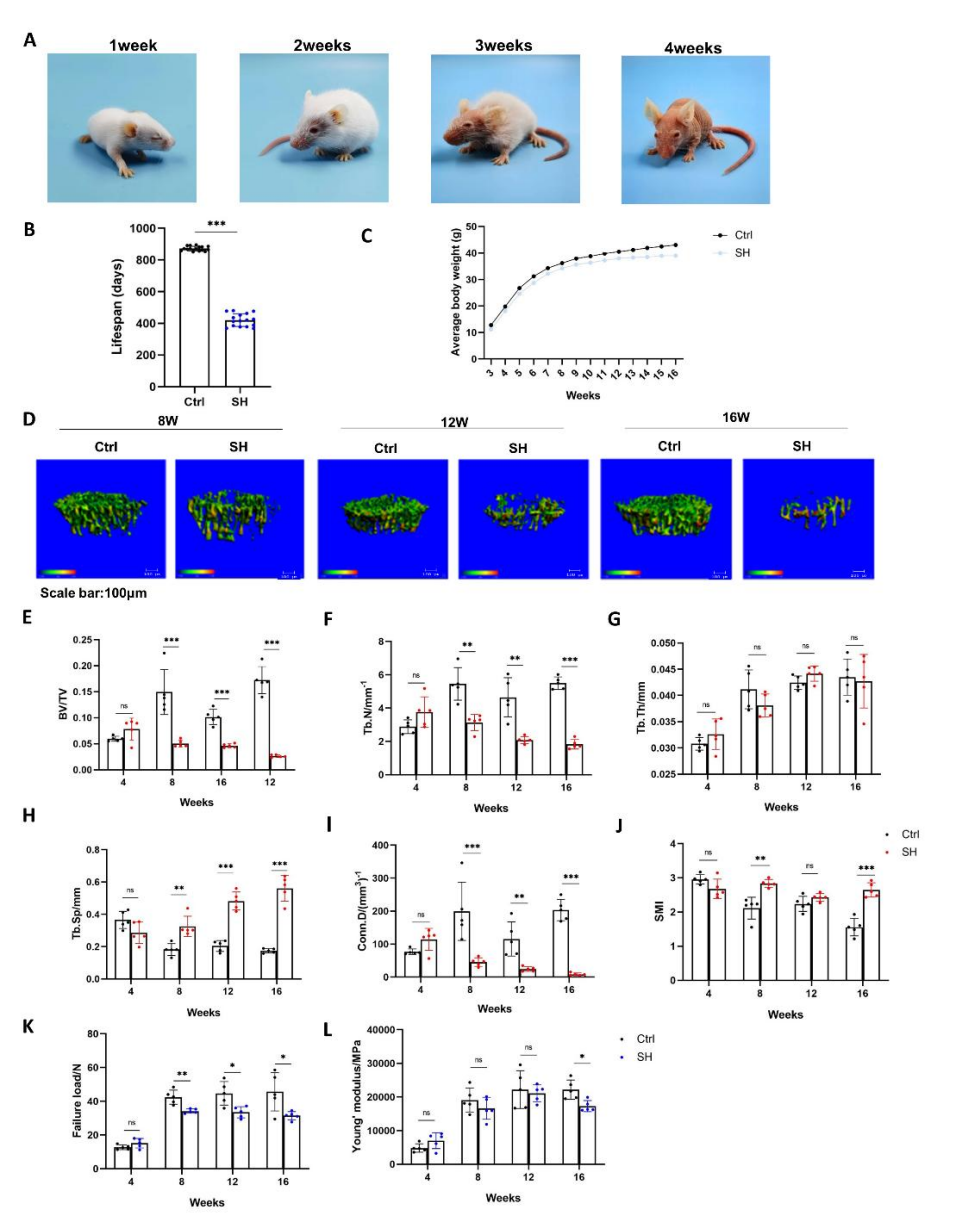
**SHJH*^hr^* mice show a significant decrease in bone mass and femoral mechanical properties after 8 weeks**. (**A**) Symptoms of hair loss in SHJH*^hr^* mice. (**B**) SHJH*^hr^* mice have a significantly shorter lifespan (n=16/group). ***P<0.001. Student's t test. (**C**) SHJH*^hr^* mice have lower mean body mass than control mice from 3 weeks of age to 16 weeks of age (n=16/group). (**D**) CT three-dimensional reconstruction of tibial trabecular tissue. Scale bar:100μm. (**E-J**) Statistics on trabecular parameters, including bone volume fraction (BV/TV), trabecular number (Tb.N), trabecular thickness (Tb.Th), connection density (Conn.D), and trabecular separation (Tb.Sp) (n=5/group, one technical replicate of five biological replicates for each group). **P<0.01, ***P<0.001. Student's t test. Non-parametric Mann-Whitney U test for 8w group of Tb.Sp. (**K**) Statistical failure loads in three-point bending tests (n=5/group, one technical replicate of five biological replicates for each group). *P<0.05, **P<0.01. Student's t test. (**L**) Statistical Young's modulus in three-point bending tests (n=5/group, one technical replicate of five biological replicates for each group). *P<0.05, **P<0.01. Student's t test. Non-parametric Mann-Whitney U test for 16w group.

Initially, we selected prematurely aging mice as the experimental animal model, sourced from an ICR hairless mouse (SHJH*^hr^*) breeding colony established by the Shanghai Jihui Laboratory Animal Care Co., Ltd [14]. Notably, SHJH*^hr^* mice began shedding hair from the tip of their nose when they were one-week-old, with complete hair loss by 4 weeks of age (Figure 1A). Preliminary lifespan analysis indicated that the average survival span of SHJH*^hr^* mice was approximately 420 days (ranging between 360-480 days), which was nearly half the lifespan of normal ICR (control) mice. The ICR mice typically live for approximately 850-900 days (Figure 1B). Furthermore, between 4 and 16 weeks of age, the body mass of SHJH*^hr^* mice was consistently lower than that of control mice (Fig. 1C).

**Figure 2.**
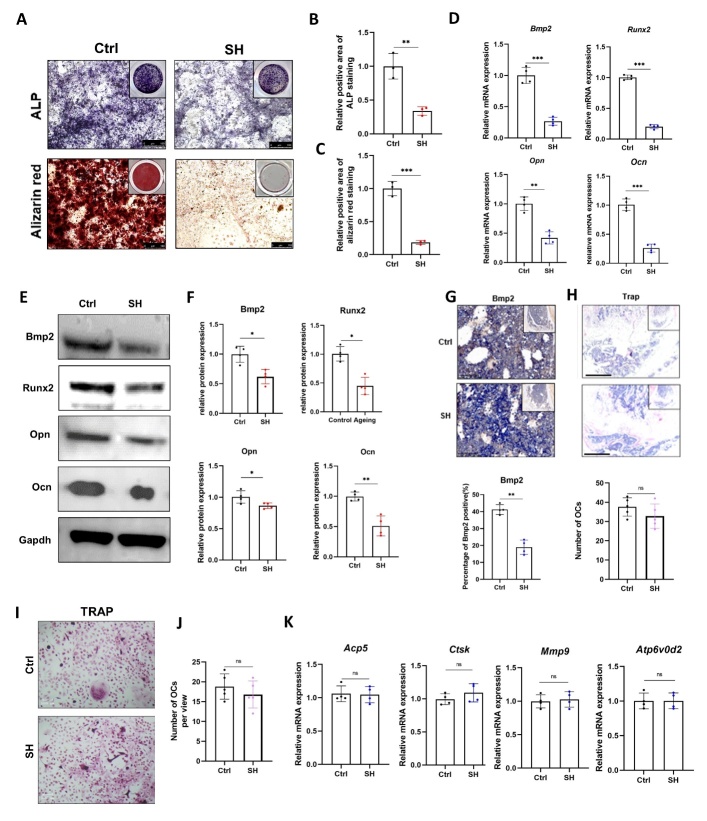
**BMSCs from SHJH*^hr^* mice showed decreased osteogenic capacity**. (**A-C**) ALP and Alizarin Red Staining of Induced BMSCs and their quantification (n=3/group, one technical replicate of three biological replicates for each group). *P<0.05, **P<0.01. Student's t test, compared with Ctrl. Scale bar:100μm. (**D-F**) Detection and quantification of osteogenesis-related gene and protein expression (n=4/group, one technical replicate of four biological replicates for each group). *P<0.05, **P<0.01, normalized to Gapdh value within the sample. ***P<0. 001.Student’s t test. (**G**) Immunohistochemistry and quantification of Bmp2 in the proximal tibia (n=4/group, one technical replicate of four biological replicates for each group). **P<0.01. Student's t test. Scale bar:50μm. (**H**) TRAP staining and osteoclast count in histologic sections of the distal femur (n=4/group, one technical replicate of four biological replicates for each group). Student's t test. (**I-J**) TRAP staining and counting after induction of BMMs into osteoblasts (n=5/group, one technical replicate of five biological replicates for each group). Student’s t test. Scale bar:100μm. (**K**) Real-time qPCR analysis of osteoblast-related gene expression (n=4/group, one technical replicate of four biological replicates for each group). Normalized to Gapdh value within the sample. Student's t test.

**Figure 3.**
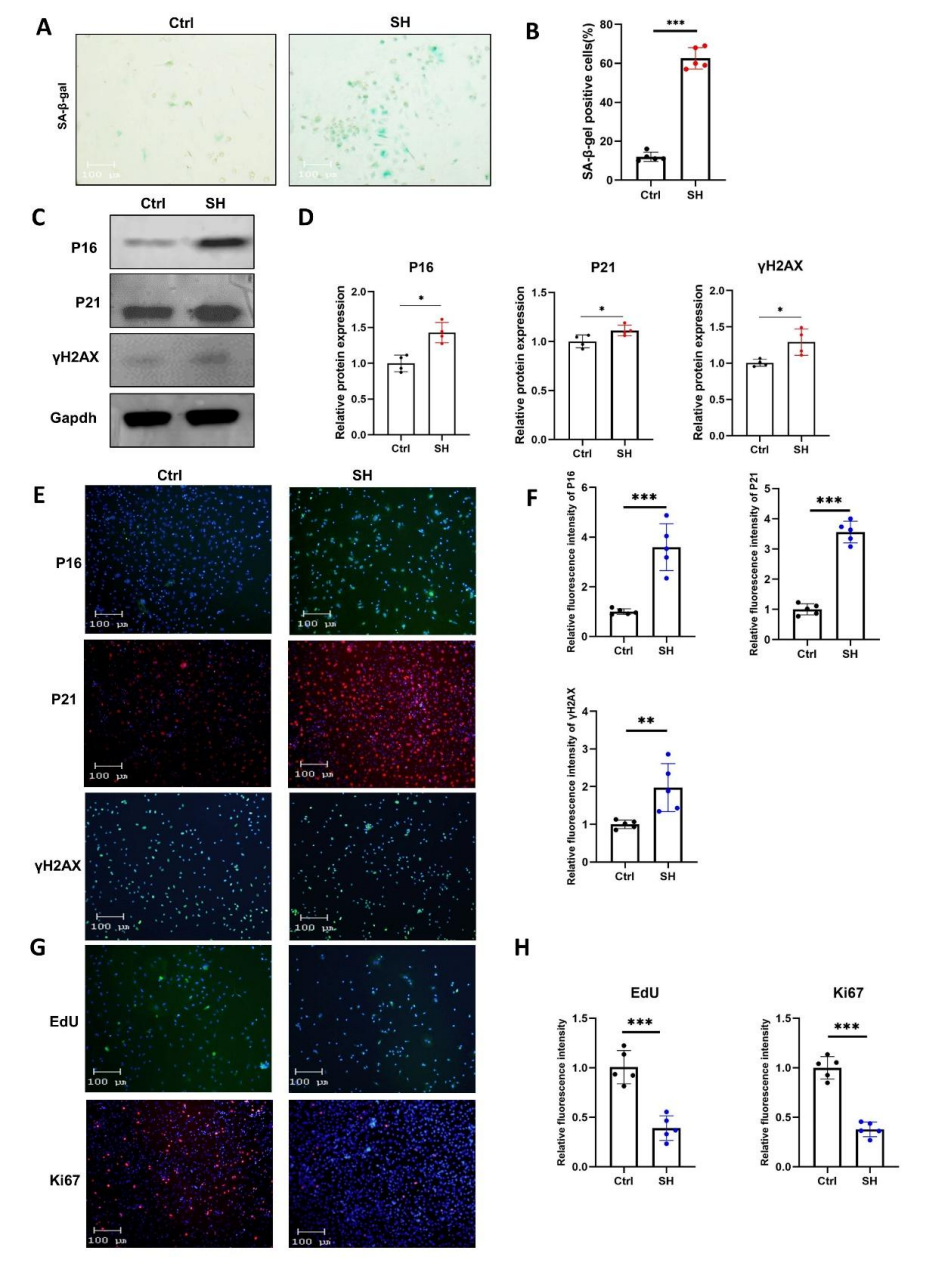
**SHJH*^hr^* mice have upregulated senescence indicators and decreased proliferation of BMSCs**. (**A-B**) β-Galactosidase staining and quantification of BMSCs (n=5/group, one technical replicate of five biological replicates for each group). **P<0.01. Student's t test. Scale bar:100μm. (**C-D**) Western blot detection of senescence indicators in BMSCs and their quantification (n=4/group, one technical replicate of four biological replicates for each group). *P<0.05. Student's t test. (**E-F**) Immunofluorescence detection of senescence indicators in BMSCs and its quantification (n=5/group, one technical replicate of five biological replicates for each group). **P<0.01, ***P<0.001. Student’s t test. Scale bar:100μm. Compared with Ctrl. (**G-H**) Proliferative capacity of BMSCs detected by EdU assay and Ki67 immunofluorescence and its quantification (n=5/group, one technical replicate of five biological replicates for each group). **P<0.01. Student’s t test. Scale bar:100μm. Compared with Ctrl.

To assess the skeletal parameters of SHJH*^hr^* mice, hind limb samples were collected from male mice at 4, 8, 12, and 16 weeks of age. Micro-CT scans of the tibia revealed a marked reduction in bone mass in SHJH*^hr^* mice compared with that in control mice after 8 weeks of age (Fig. 1D). This reduction was evident through a decrease in the cancellous bone volume fraction (BV/TV), trabecular number (Tb.N), trabecular thickness (Tb.Th), and junction density (Conn.D) and an increase in trabecular separation (Tb.Sp) (Fig. 1E-I). BV/TV (66.4%, 54.4%, and 84.8%), Tb.N (42.5%, 55.3%, and 66.8%), and Conn.D (77.3%, 78.7%, and 95.8%) of SHJH*^hr^* mice decreased significantly compared with that of the control mice, whereas Tb.Sp increased significantly (78.5%, 136.4%, and 222.1%), in three age groups(8, 12, and 16 weeks)(Fig. 1E-I). Additionally, an increase in the structural pattern index (SMI) of SHJH*^hr^* mice suggested an increase in rod-like trabeculae (Fig. 1J). Furthermore, the maximum bending load of SHJH*^hr^* mice after 8 weeks of age exhibited a significant decrease, with a notable reduction in Young's modulus after 16 weeks of age (Fig. 1K-L). In summary, SHJH*^hr^* mice displayed a marked prematurely aging phenotype with bone loss after 8 weeks of age.

### Osteogenic capacity reduced and senescence indicators increased in BMSCs from SHJH^hr^ mice

Given that osteoporosis stems from an imbalance between osteoblast-mediated bone formation and osteoclast-mediated bone resorption [16], our initial focus was to investigate the osteogenic differentiation capacity of BMSCs in SHJH*^hr^* mice. We induced osteogenic differentiation in the BMSCs isolated from 8-week-old mice. Both alkaline phosphatase and alizarin red staining indicated decreased osteogenic ability of BMSCs from SHJH*^hr^* mice (Fig. 2A-C). Post-induction, we examined the mRNA and protein expression levels of osteogenesis-related markers, including bone morphogenetic protein 2 (*Bmp2*), runt-related transcription factor 2 (*Runx2*), osteopontin (*Opn*), and osteocalcin (*Ocn*) (Fig. 2D-F). These assessments demonstrated that the osteogenic capacity of SHJH*^hr^* mice reduced significantly compared to that of control mice. Additionally, immunohisto-chemistry revealed a decrease in BMP2 levels in the proximal tibia of SHJH*^hr^* mice (Figure 3G). We also examined the number of osteoclasts in the histological sections of the distal femur and found no significant differences between the two groups (Figure 3H). We compared the osteogenic differentiation capacity of bone marrow-derived macrophages (BMM) in both mouse groups. Tartrate resistant acid phosphatase (Trap) staining showed no significant difference between the two groups (Fig. 2I-J). The results of the osteoclast-related gene expression assay supported this conclusion (Fig. 2K). These results suggest that the decreased bone mass in SHJH*^hr^* mice stems primarily from the reduced osteogenic capacity of BMSCs.

Considering the senescent phenotype observed in SHJH*^hr^* mice (Fig. 1) and the known association of decreased osteogenic capacity as a hallmark of senescence in BMSCs, we hypothesized that BMSCs in SHJH^hr^ mice would experience premature senescence compared to those in control mice. Therefore, we examined the senescence indicators of BMSCs in SHJH*^hr^* mice. As anticipated, β-galactosidase was upregulated in BMSCs of SHJH*^hr^* mice (Fig. 3A-B), and the protein expression levels of key senescence factors P16 and P21 were elevated (Fig. 3C-D). Furthermore, γH2AX was upregulated in BMSCs of SHJH*^hr^* mice, indicating a higher degree of DNA damage [17] (Fig. 3C-D). The immunofluorescence results were consistent with the western blotting findings (Fig. 3E-F). Simultaneously, Ki67 and EDU staining revealed a decreased proliferation rate of BMSCs in SHJH*^hr^* mice (Fig. 3G-H).

### Dnmt3b/l was upregulated, and Tet1 was downregulated in BMSCs of SHJH^hr^ mice

**Figure 4.**
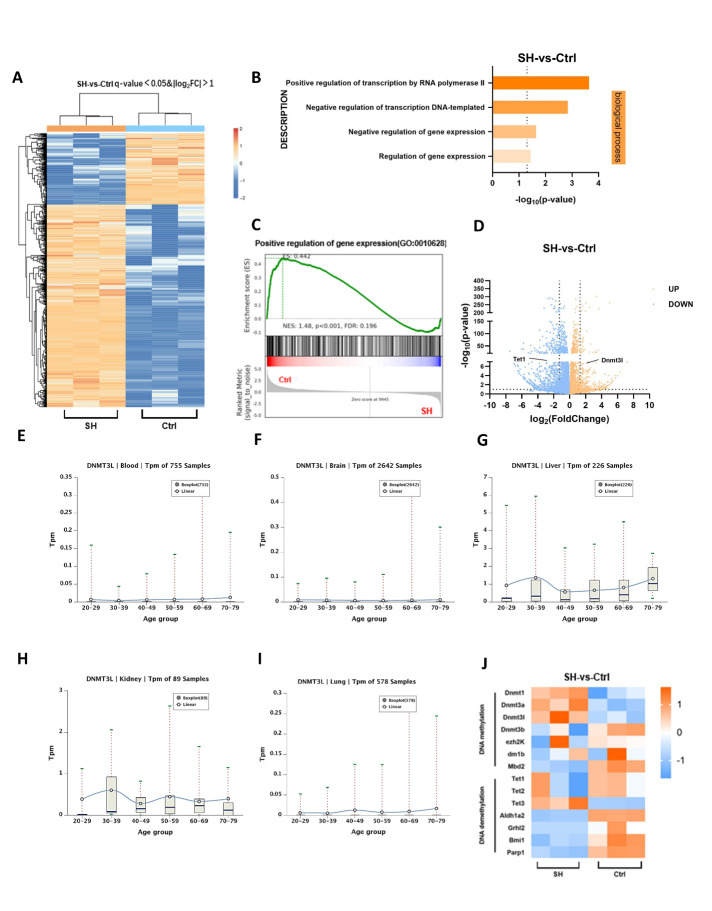
**RNA-Seq shows upregulation of Dnmt3l and downregulation of Tet1 in BMSCs from SHJH*^hr^* mice (A) Heatmap of differential gene expression**. (**B**) GO analysis of differential genes. (**C**) GESA analysis of positive regulatory pathways of gene expression. (**D**) Volcanic map of differential genes. (**E-I**) Trends of DNMT3L in human tissues with age. (**J**) Heatmap of DNA methylation/demethylation-related genes.

To determine the specific cause of reduced osteogenic capacity observed in BMSCs from SHJH*^hr^* mice, we isolated BMSCs from both groups of mice for RNA-Seq analysis. As illustrated in Fig. 4A, the results indicated significant disparities in the gene expression profiles between the two cell groups. GO enrichment analysis of the differentially expressed genes revealed significant enrichment of pathways associated with gene regulation (Fig. 4B). Concurrently, GSEA revealed enrichment of genes related to the positive regulation of gene expression pathways in the control group, whereas this enrichment was minimal in SHJH*^hr^* mice (Fig. 4C). Subsequent investigations revealed considerable upregulation of *Dnmt3l* and significant downregulation of *Tet1* in SHJH*^hr^* mice (Fig. 4D). *Dnmt3l*, a member of the DNA methyltransferase (*Dnmt*) family, regulates *Dnmt3a/b*-mediated DNA methylation [18]. In contrast, *Tet1*, a member of the ten-eleven translocation enzyme (*Tet*) family, acts as a demethylase responsible for DNA demethylation [10]. We inferred that this pattern may also be manifested in natural aging. Subsequently, we sequenced BMSCs from naturally induced SHJH*^hr^* mice, revealing a similar trend of increased *Dnmt3l* and decreased *Tet1* levels compared to those in control mice (Supplementary Fig. 1A). Additionally, we analyzed the expression patterns of DNMT3L and TET1 in human tissues using the ADEIP database (http://gb.whu.edu.cn/ADEIP/) (Fig. 4E-I, Supplementary Fig. 1B). Surprisingly, DNMT3L levels displayed an age-related increase in human tissues (blood, brain, kidney, liver, and lungs) (Fig. 4E-I). Thus, our premature aging model was consistent with the natural aging model.

**Figure 5.**
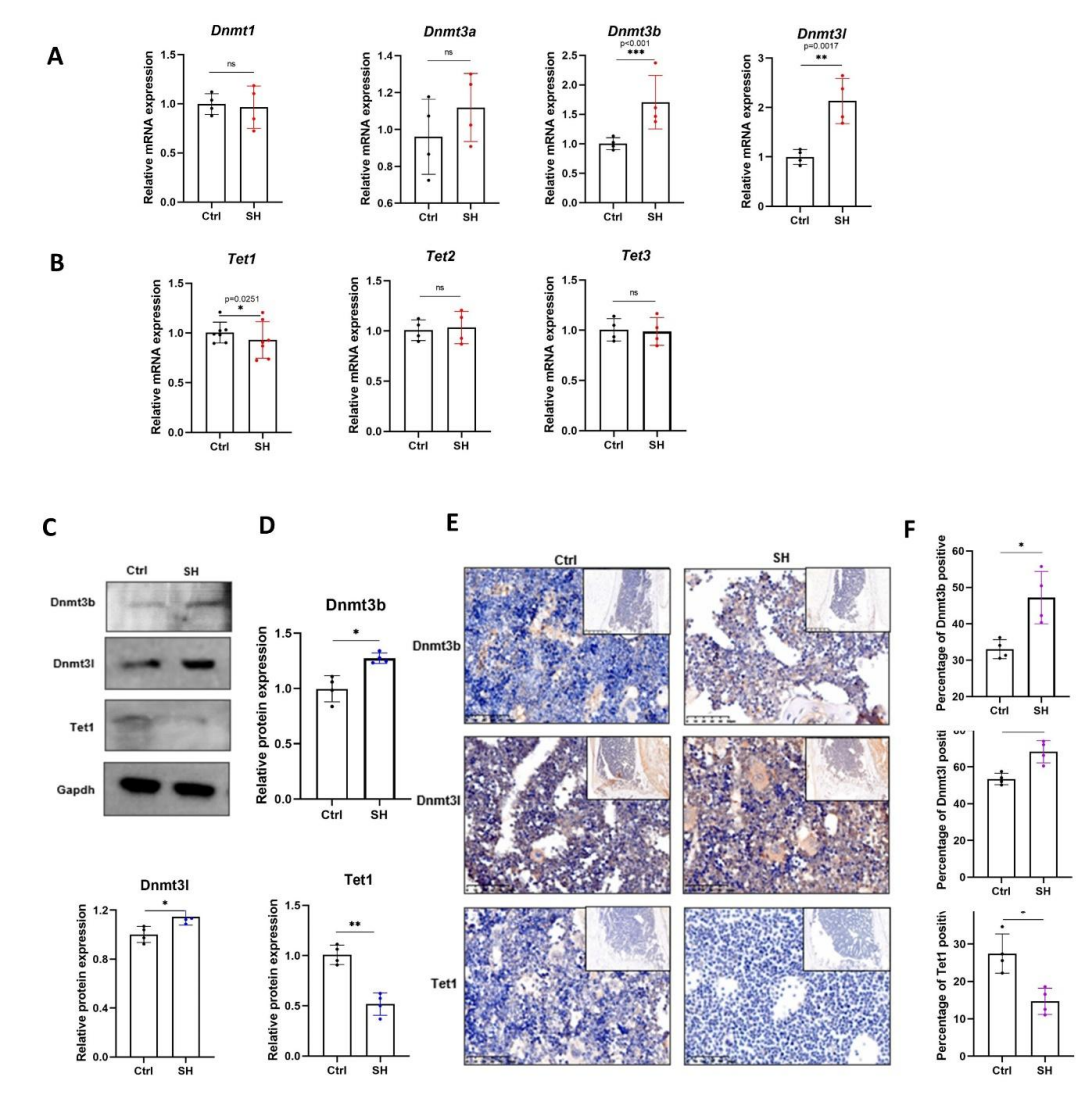
**Detection of DNA methyltransferase and DNA demethylase expression**. (**A-B**) Real-time qPCR analysis of Dnmts and Tets (n=4/group, one technical replicate of four biological replicates for each group). *P<0.05, **P<0.01, ***P<0.001, Student's t test. Normalized to Gapdh value within the sample. (**C-D**) Western blot detection of Dnmt3b/l and Tet1 expression (n=4/group, one technical replicate of four biological replicates for each group). *P<0.05, **P<0.01, Student's t test. (**E-F**) Immunohistochemistry of Dnmt3b/l and Tet1 in the proximal tibia (n=3/group, one technical replicate of three biological replicates for each group). *P<0.05, Student's t test. Scale bar:100μm.

Furthermore, we observed a general trend in methylation/demethylation-related genes. Some methylation-related genes were upregulated in SHJH*^hr^* mice, whereas certain demethylation-related genes were downregulated (Fig. 4J). Drawing from the RNA-Seq results, we examined the mRNA expression of members of the *Dnmt* and *Tet* families in the two cell groups. In addition to the differences observed in *Dnmt3l* and *Tet1*, we noted a significant upregulation of *Dnmt3b* in SHJH*^hr^* mice (Fig. 5A-B). Using the Rt-PCR results, we evaluated the protein levels of Dnmt3b, Dnmt3l, and Tet1, corroborating the trends observed in mRNA expression (Fig. 5C-D). Similarly, immunohistochemistry demonstrated the upregulation of Dnmt3b and Dnmt3l, along with the downregulation of Tet1 in SHJH*^hr^* mice (Fig. 5E-F). Collectively, we propose that DNA methylation levels in BMSCs from SHJH^hr^ mice may have been upregulated, which may have contributed to the decreased osteogenic ability.

**Figure 6.**
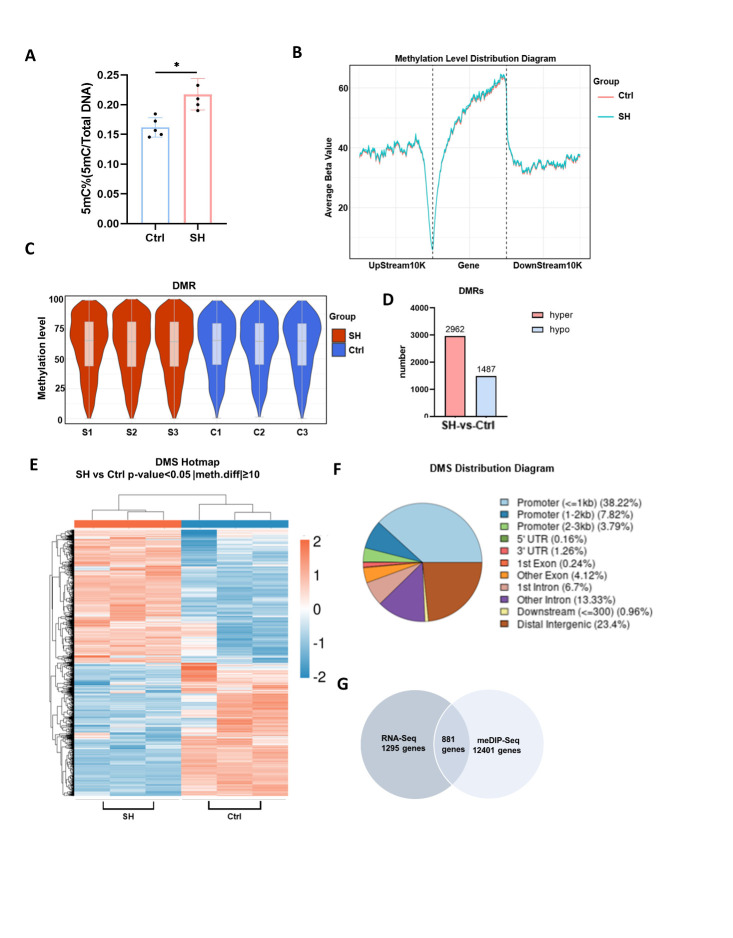
**Methylation sequencing of BMSCs**. (**A**) ELISA detection of 5mc levels in BMSCs (n=4/group, one technical replicate of four biological replicates for each group). *P<0.05, Student's t test. (B) The distribution plot of the average methylation level at each site in the upstream and downstream 10-kb region of the gene transcription start site (TSS). (**C**) Violin plots depicting the density of methylation percentages in differentially methylated regions (DMRs). (**D**) Compared to the Control group, DMRs were returned in the SHJH*^hr^* mice. False Discovery Rate (FDR) < 0.05 and CpG number ≥ 5. (**E**) Heat maps of differentially methylated sites (DMSs). (**F**) Percentage of DMS in different gene functional regions. (**G**) Venn plots of DMS-related and differentially expressed genes.

**Figure 7.**
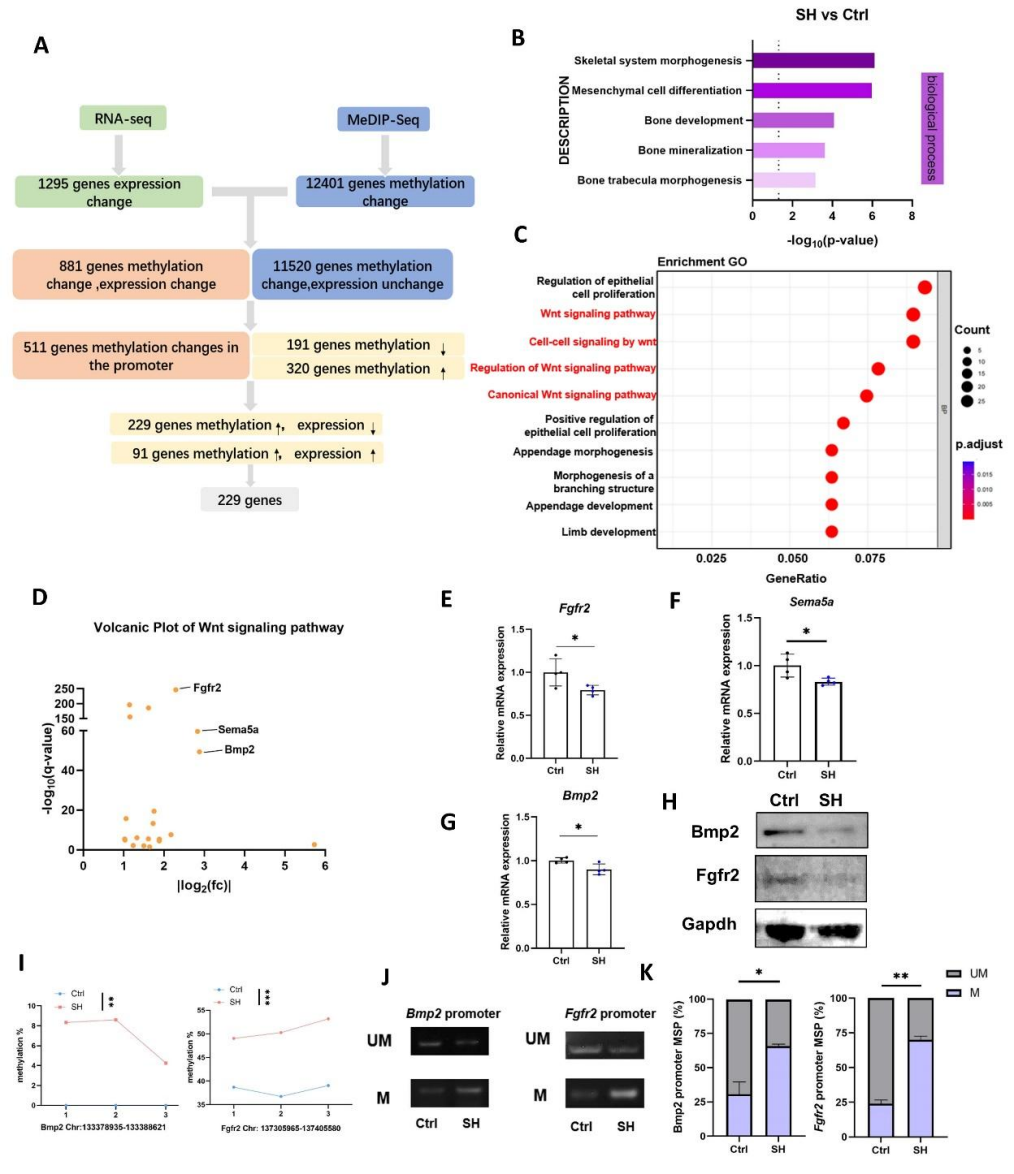
**Combined screening to identify target signaling pathways and genes.(A) Combined analysis of RNA-Seq and MeDIP-Seq.(B) GO analysis enriches signaling pathways related to MSC differentiation and bone growth and development.(C) Bubble plots of the top ten signaling pathways in the GO analysis.(D) Volcano map of genes in the Wnt signaling pathway.(E-G) Real-time qPCR analysis of Bmp2,Fgfr2 and Sema5a (n=4/group, one technical replicate of four biological replicates for each group)**. *P<0.05, **P<0.01, Student's t test. Normalized to Gapdh value within the sample. (**H**) Western blot detection of protein levels of Bmp2 and Fgfr2 (n=4/group, one technical replicate of four biological replicates for each group). (**I**) Visualization of methylation levels of Bmp2 and Fgfr2 (n=3/group). **P<0.01, ***P<0.001, Student’s t test. (**J-K**) MSP Detection of Methylation Levels in the Promoter Regions of Bmp2 and Fgfr2 (n=3/group, one technical replicate of three biological replicates for each group). *P<0.05, Student's t test. MSP primers were designed for sites (Bmp2:133378745, Fgfr2:137404048) from methylation sequencing. The methylated forward primer sequence for Bmp2 was TTGAGTAGGGGGAGGAGTGCG, and the unmethylated forward primer was TTGAGTAGGGGGAGGAGTGTG. The reverse primer sequences were both ACCCCAAAC CATAAATCACAACCCAA. the methylated forward primer sequence for Fgfr2 was TATGAGGTGTGTGTT TAATATATAGTTTTACG, and the unmethylated forward primer was TATGAGGTGTGTGTTTAAT ATATAGTTTTATG. the reverse primer sequences were all CCCCTCACATTAAACCCTT.

### DNA methylation levels were upregulated in BMSCs of SHJH^hr^ mice

Initially, we assessed 5mC levels in the cellular DNA of both experimental groups. Comparative analysis revealed an upregulation in the level of 5mC in BMSCs of SHJH*^hr^* mice compared to that in the control group (Fig. 6A). This observation strongly suggested a potential upregulation in the DNA methylation levels of BMSCs in SHJH*^hr^* mice, which was consistent with previous findings [19]. Subsequently, we focused on unraveling DNA methylation alterations in SHJH*^hr^* mice using methylation capture sequencing (MeDIP-seq). The distribution plot showing the average methylation level at each site within the 10 kb region upstream or downstream of the gene transcription start site (TSS) depicted a consistently higher curve in SHJH*^hr^* mice than in the control group. This indicated increased methylation of the promoter regions in SHJH*^hr^* mice genes (Fig. 6B). In addition, we concentrated on differentially methylated regions (DMRs) and differentially methylated sites (DMSs). The violin plot of DMRs displayed a higher average methylation level of CpGs in SHJH*^hr^* mice than those in the control group (Fig. 6C). After screening based on the criteria of false discovery rate (FDR) < 0.05 and CpG number ≥ 5, we identified 4449 DMRs, comprising 2962 significantly hypermethylated and 1487 hypomethylated DMRs, in SHJH*^hr^* mice, as compared to those in the control group (Fig. 6D). Furthermore, the heatmap representation of DMSs demonstrated a higher number of hypermethylated sites in SHJH*^hr^* mice than in control mice (Fig. 6E). Considering the diverse ways in which gene expression is regulated after methylation across various gene regions, we tabulated the number of DMSs in the different functional regions. Notably, the majority of DMSs were distributed within the promoter regions of the genes (49.83%) (Fig. 6F). This prompted a deeper focus on genes with methylated loci in their promoter regions. Subsequently, we identified 881 genes associated with DMS that were also differentially expressed (Fig. 6G).

### Combined screening revealed hypermethylation in the promoter regions of Bmp2 and Fgfr2 genes in BMSCs from SHJH^hr^ mice

To probe the correlation between the diminished osteogenic differentiation potential of SHJH*^hr^* mouse BMSCs and their genomic hypermethylation, we conducted a combined screening using RNA sequencing and methylation sequencing. Among the 881 genes identified by intersecting the results of RNA-Seq and MeDIP-seq, we specifically focused on 551 genes in which DMSs were present in the promoter regions. Although some genes displayed both hyper-and hypomethylated sites in the promoter region, the overall count revealed 191 genes with hypomethylated sites and 320 genes with hypermethylated sites in the promoter region. Given the upregulated DNA methylation levels observed in BMSCs of SHJH*^hr^* mice, our primary focus was on genes exhibiting hypermethylated sites in their promoter regions. Typically, elevated methylation levels in the promoter regions correlate with decreased gene expression [20]. We identified 229 genes with hypermethylated sites in their promoter regions and decreased gene expression (Fig. 7A).

Subsequently, we subjected these genes to GO analysis and noted significant enrichment in the signaling pathways associated with MSC differentiation, bone growth, and development (Fig. 7B). Notably, among the top ten signaling pathways, the Wnt signaling-related pathway was prominently enriched (Figure 7C). Consequently, we focused on genes within the Wnt signaling pathway, and *Bmp2, Fgfr2,* and *Sema5a* exhibited the most noteworthy changes (Fig. 7D). Quantitative Real-time PCR showed that *Bmp2, Fgfr2* and *Sema5a* were significantly decreased in BMSCs from SHJHhr mice (Figure 7E-G). *Bmp2* and *Fgfr2* are closely associated with the osteogenic differentiation of BMSCs [21, 22]. *Bmp2* can induces directed differentiation and proliferation of undifferentiated MSCs into chondrocytes and osteoblasts, promoting the differentiation and maturation of osteoblasts, which are crucial for bone growth, development, and reconstruction [21]. Similarly, *Fgfr2*, a pivotal factor in osteogenic differentiation, stimulates osteoblast proliferation and differentiation while inhibiting senescence [22]. Therefore, we prioritized *Bmp2* and *Fgfr2* for further investigation. Consistent with the results of Quantitative Real-time PCR, a significant decrease in Bmp2, Fgfr2 protein expression was also observed in BMSCs from SHJH*^hr^* mice (Fig. 7H). Visualization of methylation levels of representative CpG sites in the promoter regions of *Bmp2* and *Fgfr2* revealed elevated methylation levels in SHJH*^hr^* mice compared to those in the control group (Fig. 7I). Additionally, using methylation-specific PCR (MSP), we confirmed hypermethylation of the promoter regions of *Bmp2* and *Fgfr2* in SHJH*^hr^* mice (Fig. 7J-K). These findings substantiate the notion that the diminished osteogenic differentiation ability observed in BMSCs of the SHJH*^hr^* mice may be attributed to aberrant hypermethylation within the promoter regions of *Bmp2* and *Fgfr2*.

**Figure 8.**
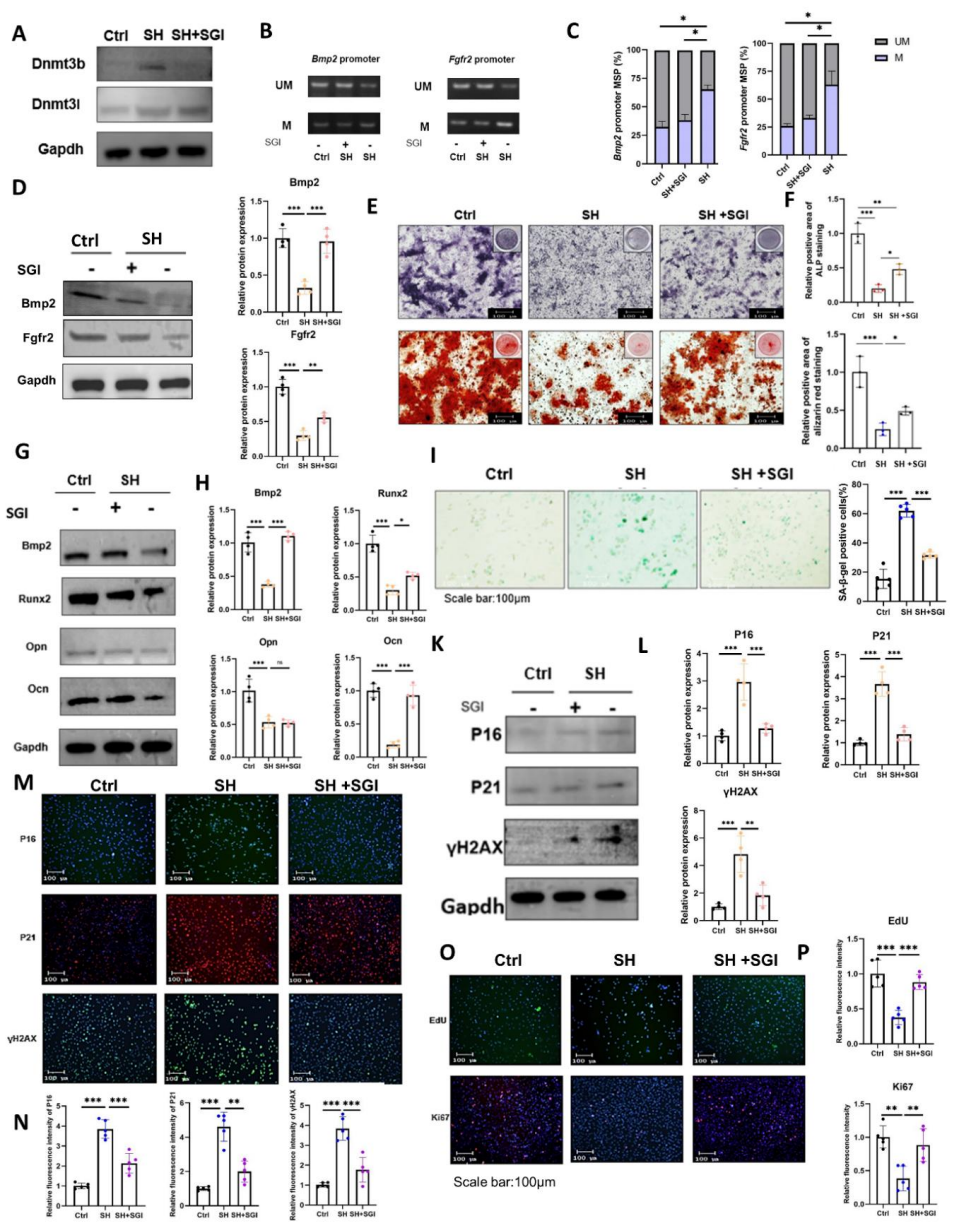
**SGI-1027 rescues the osteogenic differentiation potential of BMSCs in SHJH*^hr^* mice**. (**A**) Western blot detection of protein levels of Dnmt3b/l (n=4/group, one technical replicate of four biological replicates for each group). (**B**) MSP Detection of Methylation Levels in the Promoter Regions of Bmp2 and Fgfr2 (n=3/group, one technical replicate of three biological replicates for each group). *P<0.05, one-way ANOVA. (**C-D**) Western blot detection of protein levels of Bmp2 and Fgfr2 (n=4/group, one technical replicate of four biological replicates for each group). *P<0.05, one-way ANOVA. (**E-F**) ALP and Alizarin Red Staining of Induced BMSCs and their quantification (n=3/group, one technical replicate of three biological replicates for each group). *P<0.05, **P<0.01, one-way ANOVA. Scale bar:100μm. Compared with Ctrl. (**G-H**) Detection and quantification of osteogenesis-related protein expression (n=4/group, one technical replicate of four biological replicates for each group). *P<0.05, ***P<0.001. one-way ANOVA. (**I-J**) β-Galactosidase staining and quantification of BMSCs (n=5/group, one technical replicate of five biological replicates for each group). Scale bar:100μm, *P<0.05, ***P<0.001. one-way ANOVA. (**K-L**) Western blot detection of senescence indicators in BMSCs and their quantification (n=4/group, one technical replicate of four biological replicates for each group). *P<0.05, ***P<0.001. one-way ANOVA. (**M-N**) Immunofluorescence detection of senescence indicators in BMSCs and their quantification (n=5/group, one technical replicate of five biological replicates for each group). **P<0.01, ***P<0.001. one-way ANOVA. Scale bar:100μm. (**O-P**) Proliferative capacity of BMSCs detected by EDU assay and Ki67 immunofluorescence and their quantification (n=5/group, one technical replicate of five biological replicates for each group). *P<0.05, **P<0.01, one-way ANOVA.Scale bar:100μm.

### Methyltransferase inhibitor can rescue bone mass in SHJH^hr^ mice

To validate our hypothesis, we treated BMSCs from SHJH*^hr^* mice with SGI-1027, an inhibitor of DNA methyltransferase [23]. Initially, we detected the expression level of Dnmt3b/l after using inhibitors. Our results show that SGI-1027 directly inhibits Dnmt3b.Dnmt3l lacks conserved catalytic residues and most relys on Dnmt3a or Dnmt3b to function [24, 25]. Therefore, SGI-1027 indirectly affects Dnmt3l (Fig. 8A). And then, we conducted a cytotoxicity assay and determined 4 μmol/L as the optimal experimental concentration (Supplementary Fig. 2). Subsequent treatment with SGI-1027 notably reduced the methylation levels within the promoter regions of *Bmp2* and *Fgfr2*, while concurrently restoring their protein expression levels (Fig. 8B-D).

**Figure 9.**
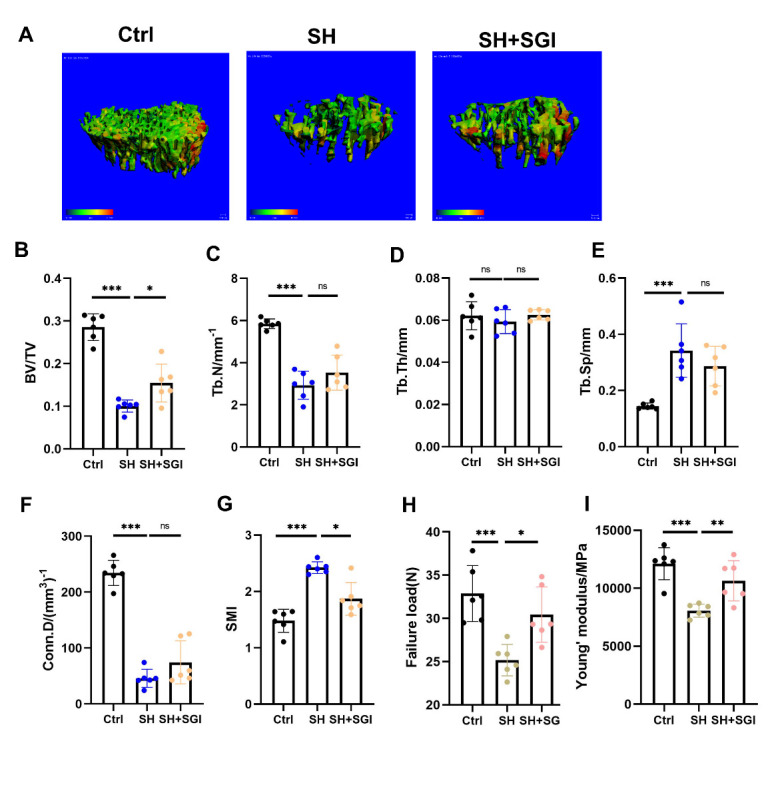
**SGI-1027 increased bone mass in SHJH*^hr^* mice**. (**A**) CT three-dimensional reconstruction of tibial trabecular tissue. Scale bar:100μm. (**B-G**) Statistics on trabecular parameters, including bone volume fraction (BV/TV), trabecular number (Tb.N), trabecular thickness (Tb.Th), connection density (Conn.D), and trabecular separation (Tb.Sp) (n=6/group, one technical replicate of six biological replicates for each group). **P<0.01, ***P<0.001. Student's t test. (**H-I**) Statistical failure loads and Young's modulus in three-point bending tests (n=6/group, one technical replicate of six biological replicates for each group). *P<0.05, **P<0.01, ***P<0.001. Student's t test.

Remarkably, SGI-1027 treatment rescued the osteogenic differentiation capacity of BMSCs in SHJH*^hr^* mice. Compared with the untreated SH group, significant improvement in the osteogenic differentiation of BMSCs was observed in the SH+SGI group (Figure 8E-H). To verify the methylation level of the Bmp2 and Fgfr2 is closely connected with the osteogenic differentiation capacity of BMSCs in the SHJH*^hr^* mice, we used siRNA to knock down Bmp2 and Fgfr2. Both ALP and Alizarin Red staining showed that knocking down Bmp2 and Fgfr2 reduced the salvage effect of SGI-1027(Supplementary Fig. 3A-B). The detection results of osteogenic markers (Bmp2, Runx2, and Ocn) also confirmed the conclusion (Supplementary Fig. 3C-D). This suggests that SGI-1027 is difficult to function in the absence of Bmp2 and Fgfr2. Additionally, SGI-1027 treatment reduced the β-galactosidase content and decreased the levels of P16, P21, and γH2AX in BMSCs derived from SHJH*^hr^* mice (Fig. 8I-L). Notably, immunofluorescence showed that SGI-1027 reduced the levles of P16, P21, and γH2AX and promoted the proliferation of BMSCs in SHJH*^hr^* mice (Fig. 8M-P).

**Figure 10.**
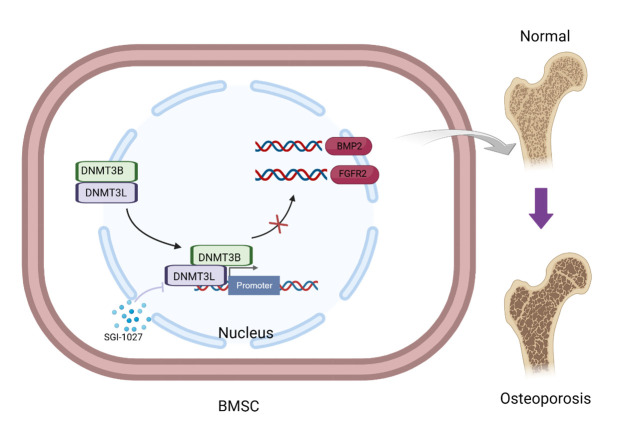
Mechanistic map of Dnmt3b/l regulation of osteogenic differentiation of BMSCs.

The results of micro-CT showed that SGI-1027 significantly increased bone mass in SHJH*^hr^* mice (Fig. 9A) compared to the SH group. SGI-1027 treated mice showed a significant increase in BV/TV (Fig. 9B-G). Similarly, the results of the three-point bending test showed that SGI-1027 significantly increased the mechanical properties of SHJH*^hr^* mice (Fig. 9H-I). In conclusion, inhibition of methyltransferase has a therapeutic effect on increaing bone mass in SHJH*^hr^* mice (Fig 10).

## DISCUSSION

The significant influence of epigenetic modifications on the function and expression of genes associated with osteoporosis has been demonstrated [26]. Deciphering the epigenetic regulation of osteoporosis is pivotal for understanding its onset and progression, potentially paving the way for novel therapeutic strategies in clinical settings.

In the present study, SHJH*^hr^* mice displayed a prematurely aging phenotype with distinct skeletal senescence. Combined analysis of the transcriptome and methylome revealed that a crucial signaling pathway, the Wnt signaling pathway, was affected by DNA methylation. Additionally, methylation capture sequencing revealed widespread genomic hyper-methylation in the BMSCs. By integrating methylome and transcriptome data, we identified several key methylated genes and networks potentially involved in osteoporosis development. Notably, elevated methylation levels of genes associated with the Wnt signaling pathway, specifically those of *Bmp2* and *Fgfr2*, were linked to decreased expression levels, compromising the osteogenic differentiation potential of BMSCs. The osteogenic effects of *Bmp2* are primarily linked to its regulation of downstream *Runx2* and *Osterix* [27]. *Fgfr2* activates Wnt/β-catenin signaling, thus fostering osteoblast differentiation, suppressing adipocyte differentiation, and enhancing *Runx2* expression [28]. Moreover, *Fgfr2* has been reported to enhance MSC proliferation and deter senescence [28], potentially explaining SGI-1027's ability to counter the senescent phenotype of BMSCs. Overall, our findings offer novel insights into the role of DNA methylation in osteoporosis development and suggest promising avenues for exploring epigenetic interventions as potential treatment strategies for osteoporosis (Fig. 9).

In this study, we used SHJH*^hr^* mice as an animal model of aging, which displayed a significant skeletal degeneration phenotype at 8 weeks of age, much shorter than that in SMAP8 mice, which show senescence phenotype only after 6 months of age [29]. Premature aging can be caused by many factors, such as mutations in *LMNA* and altered post-translational maturation of prelamin A, which may cause accelerated aging and bone loss [30]. SHJH*^hr^* mice have a much longer lifespan than Hutchinson-Gilford premature aging mice, which live only for around 200 days [31] and can be used for skeletal senescence studies and related to skin aging [14] and hypothyroidism [32]. Premature aging has many causes, including genomic instability, cellular senescence, and epigenetic alterations. Li et al identified that SHJH*^hr^* mice have a nonsense mutation (2134 C→T) in the *Hr* gene, and the mice exhibit spontaneous hyperthyroidism in addition to hair shedding [32]. Additionally, it is necessary to study the causes of premature aging in SHJH*^hr^* mice using animal models.

Changes in DNA methylation-related enzymes have been reported in studies of osteoporosis. *Tet1* and *Tet2* deficiency reduces the demethylation of the *P2rX7* promoter and downregulates exosome release, leading to the intracellular accumulation of miRNAs that inhibit *Runx2* signaling and impair BMSC function [33]. Here, we showed a positive effect of *Tet1* on the maintenance of *Bmp2* and *Fgfr2* signaling, which suggests direct miRNA-independent regulation of the osteogenic signaling pathway by *Tet1*. In addition, Dnmt1 promotes methylation of the promoter region of osteoprotegerin (*Opg*) and inhibits its expression, which in turn impairs the osteogenic differentiation of BMSCs [34]. Combined with our data, these results suggest that *Dnmt* genesnegatively regulate the osteogenic differentiation of BMSCs. Importantly, our results suggest that the regulation of osteogenesis in BMSCs by the *Dmnt* family is not limited to Opg signaling, and that different target genes may exist for different *Dnmt* genes. In summary, our study unearthed *Dnmt3b*/*l*, two negative regulators of osteogenesis that have not been reported previously. The inhibition of the Wnt signaling pathway by DNMT3B/L was remarkable. By analyzing the genes related to the Wnt signaling pathway, we identified two key genes, *Bmp2* and *Fgfr2*, that are targeted by DNMT3B/L. DNMT3B/Lbinds to the promoter regions of *Bmp2* and *Fgfr2* and reduces their expression, leading to the suppression of BMSC osteogenic differentiation. Our findings confirm the direct regulatory role of DNA methylation in Bmp2 and Fgfr2 signaling, further broadening the mechanism by which DNA methylation regulates the osteogenic differentiation of BMSCs. However, this study has some limitations. As mentioned above, mutations in the *Hr* gene are present in the skin cells of SHJH*^hr^* mice. However, we cannot rule out the potential effects of these gene mutations. Similarly, for epigenetic variations, we focused only on DNA methylation and ignored other possibilities (such as histone modification and non-coding RNA regulation). Other epigenetic variants may also contribute to bone loss in SHJH*^hr^* mice. For example, histone epitope modifications have an important role in bone reconstruction and bone development [35]. Uncovering other moderating factors should be the focus of subsequent studies.

In conclusion, our findings underscore that aberrant upregulation of *Dnmt3b/3l* and downregulation of *Tet1*, leading to the hypermethylation and inhibition of the key osteogenic factors *Bmp2* and *Fgfr2*, impair the osteogenic differentiation potential of BMSCs. This novel epigenetic regulatory pathway is depicted as a mechanistic picture and offers insights into potential osteoporosis-associated mechanisms. Although synthetic small-molecule DNA demethylating agents may not be ideal for prophylactic use in osteoporosis owing to widespread DNA demethylation and potential cytotoxicity, our results lay a new theoretical groundwork for epigenetic interventions in osteoporosis treatment.

## Supplementary Materials

The Supplementary data can be found online at: www.aginganddisease.org/EN/10.14336/AD.2024.0324.


