# Supplementary material for: Hypermethylation of Bmp2 and Fgfr2 Promoter Regions in Bone Marrow Mesenchymal Stem Cells Leads to Bone Loss in Prematurely Aged Mice
Source: Aging Dis. 2024 Mar 24;16(2):1149–68. doi: 10.14336/AD.2024.0324 (PMC11964434; doi:10.14336/AD.2024.0324)
Supplement: Supplementary file 1 [file AD-16-2-1149-s.pdf]

SUPPLEMENTARY DATA

Hypermethylation of *Bmp2* and *Fgfr2* Promoter Regions in Bone Marrow Mesenchymal Stem Cells Leads to Bone Loss in Prematurely Aged Mice

Yao Wang, Lin Sun, Tianyou Kan, Wendong Xue, Han Wang, Ping Xu, Lei Zhang, Mengning Yan, Hanjun Li, Zhifeng Yu

© 2024. Wang Y et al. Published online at http://www.aginganddisease.org/EN/10.14336/AD.2024.0324

SUPPLEMENTARY DATA

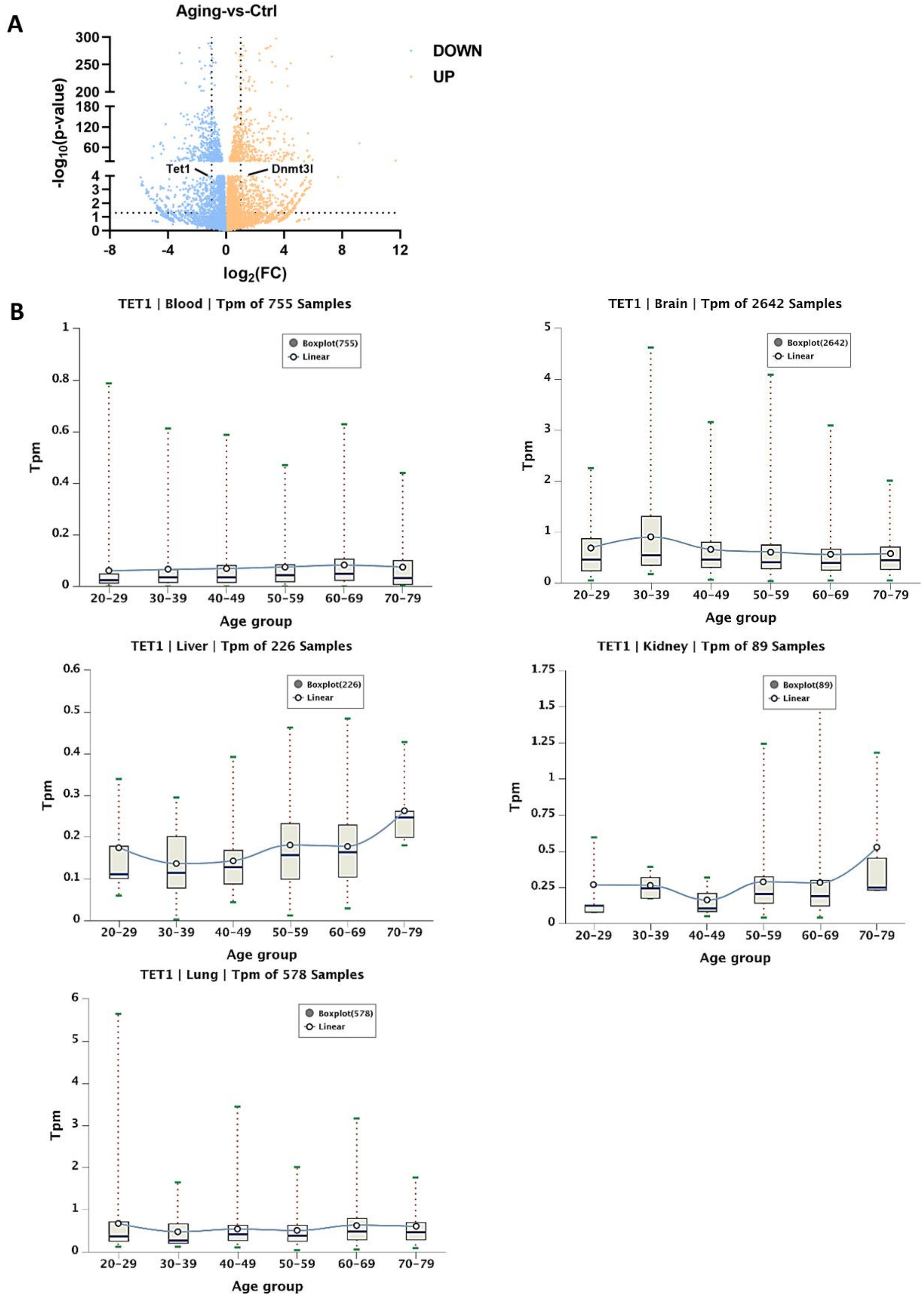

Supplementary Figure 1. Expression trends of Dnmt1 and Tet1 in naturally aging mouse BMSCs and aging human organs. (A) Volcano plots of differential genes identified by RNA-Seq between 18-month-old and 8-week-old mice. (B) Trends of *Tet1* expression in 20-79-year-old human organs.

© 2024. Wang Y et al. Published online at http://www.aginganddisease.org/EN/10.14336/AD.2024.0324

SUPPLEMENTARY DATA

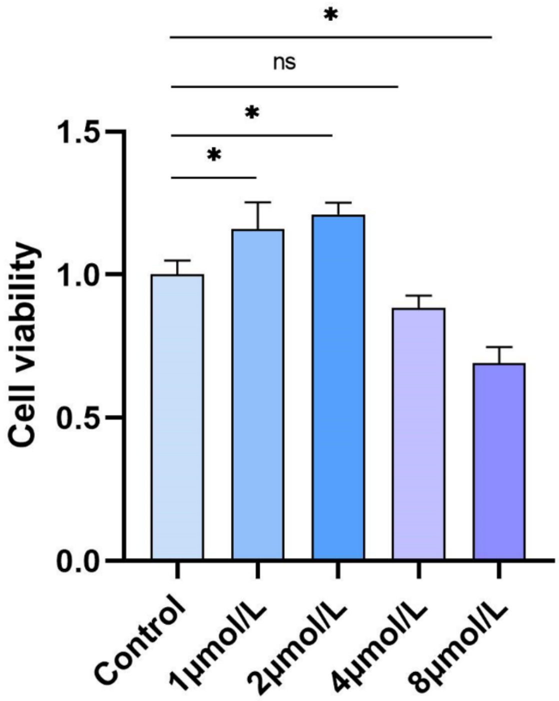

Supplementary Figure 2. **Cytotoxicity assay of SGI-1027.**

© 2024. Wang Y et al. Published online at http://www.aginganddisease.org/EN/10.14336/AD.2024.0324

SUPPLEMENTARY DATA

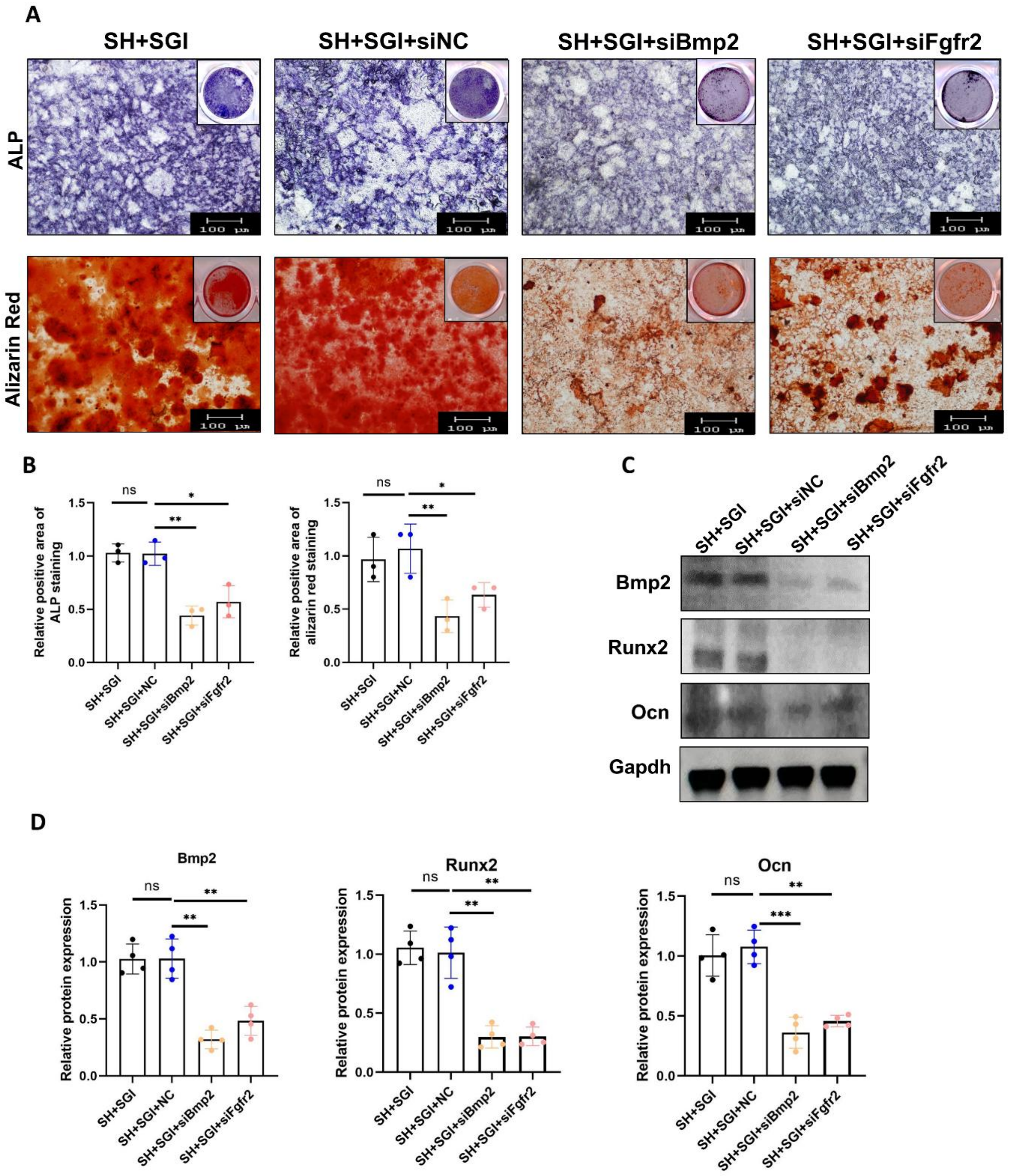

Supplementary Figure 3. **Knockdown of Bmp2 and Fgfr2 reduces the rescue effect of SGI-1027. (A)** ALP and Alizarin Red Staining of Induced BMSCs and their quantification (n=3/group, one technical replicate of three biological replicates for each group). *P<0.05, **P<0.01, one-way ANOVA. Compared with SH+SGI. For the sequence of siRNA(5'to3'), Fgfr2: UGGCUGAAGCAGUGGGAAUTT, AUUCCCACUGCUUCAGCCATT.Bmp2: CGGAAGACGUCCUCAGCGATT,UCGCUGA GGACGUCUUCCGTT.NC: UUCUCCGAACGUGUCACGUTT, ACGUGACACGUUCGGAGAATT. (**B-G)** Western blot detection of protein levels of Osteogenesis-related factors (n=4/group, one technical replicate of four biological replicates for each group) *P<0.05, **P<0.01, one-way ANOVA.

© 2024. Wang Y et al. Published online at http://www.aginganddisease.org/EN/10.14336/AD.2024.0324
